# Estimated Savings From the Medicare Shared Savings Program

**DOI:** 10.1001/jamahealthforum.2023.4449

**Published:** 2023-12-15

**Authors:** Andrew M. Ryan, Adam A. Markovitz

**Affiliations:** 1 Department of Health Services, Policy and Practice, Brown University School of Public Health, Providence, Rhode Island; 2 Department of Internal Medicine, University of Michigan, Ann Arbor

## Abstract

**Importance:**

The Medicare Shared Savings Program (MSSP) is the largest and most important alternative payment model that has been implemented by the Centers for Medicare & Medicaid Services (CMS). Its budgetary impact to CMS is not well understood.

**Objective:**

To evaluate the association between the MSSP and net savings to CMS for performance years 2013 to 2021.

**Design, Setting, and Participants:**

The economic evaluation used publicly reported data on the MSSP from April 1, 2012, to December 31, 2021, and estimates extracted from 2 prior studies.

**Main Outcomes and Measures:**

Net savings to CMS, calculated as the difference between incentive payments to MSSP accountable care organizations and gross spending reductions. Incentive payments were calculated using the publicly reported data. The association of the MSSP with gross medical spending in traditional Medicare was extracted from 2 prior studies. Spillovers of the MSSP to Medicare Advantage (MA) were estimated by evaluating how net spending changes from the MSSP impacted benchmark payments to MA plans. Net savings from traditional Medicare and MA were then combined.

**Results:**

The MSSP was associated with net losses to traditional Medicare of between $584 million and $1.423 billion over the study period. Losses from MSSP-related reductions to MA benchmarks totaled between $191 million and $640 million. Across traditional Medicare and MA, the MSSP was associated with losses of between $775 million and $2.063 billion. This represents approximately 0.028% of combined spending for traditional Medicare and MA over the study period.

**Conclusions and Relevance:**

This economic evaluation found that the MSSP was associated with net losses to CMS. The total budget impact of the MSSP to CMS was small and continues to be uncertain due to challenges in estimating the effects of the MSSP on gross spending, particularly in recent years.

## Key Points

**Question:**

Was the Medicare Shared Savings Program (MSSP) associated with net savings to the Centers for Medicare & Medicaid Services (CMS)?

**Findings:**

In this economic evaluation using estimates from 2 studies and data on MSSP incentive payments for MSSP performance years 2013 to 2021, the MSSP was associated with net losses to CMS of between $775 million and $2.063 billion.

**Meaning:**

The MSSP has resulted in a small increase to CMS spending.

## Teaser

This economic evaluation examines whether implementation of the Medicare Shared Savings Program was associated with net savings to the Centers for Medicare & Medicaid Services during performance years 2013 to 2021.

## Introduction

The Medicare Shared Savings Program (MSSP) was launched in 2012 to improve efficiency and generate financial savings for the Centers for Medicare & Medicaid Services (CMS). Under the program, voluntarily constituted accountable care organizations (ACOs)—groups of clinicians, hospitals, and other institutional participants—face accountability for the total costs of care received by traditional Medicare beneficiaries. If medical spending is below a specific target (benchmark), ACOs are eligible for financial bonuses. For CMS to break even or achieve net savings for traditional Medicare beneficiaries in the MSSP, gross reductions in medical spending must equal or exceed the sum of bonus payments paid to ACOs. Reductions in medical spending may also spill over to Medicare Advantage (MA) because MA benchmarks are based on spending in traditional Medicare.

The effect of the MSSP on net savings to CMS depends on 3 important factors. First, in traditional Medicare, gross reductions in medical spending appear to be concentrated among physician-only ACOs and not ACOs affiliated with a hospital.^1^ Second, savings to CMS depend on how MSSP benchmarks are set: benchmarks that are too easy to achieve will result in bonus payments that are too high relative to ACO performance. Third, because benchmarks in MA depend on projected national per capita traditional Medicare spending (known as the US Per Capita Cost [USPCC]) net changes to Traditional Medicare spending caused by the MSSP will affect MA benchmarks and subsequent payment to MA plans (eMethods in Supplement 1).

The most recent assessment of net savings in MSSP evaluated the 2012 to 2016 period.^2^ To our knowledge, no evaluations have incorporated how ACO bonus payments have evolved over time, particularly after the COVID-19 pandemic, and no evaluations have assessed the association of the MSSP with payment in MA. In this economic evaluation, we combined prior estimates of the association between the MSSP and gross spending in traditional Medicare with estimates of bonus payments to MSSP ACOs and new projections of how these estimates impact payments to MA plans to evaluate net changes in CMS spending in the first 9 performance years of the MSSP.

## Methods

Publicly reported data on the MSSP from April 1, 2012, to December 31, 2021, were used. This included information about ACOs’ total expenditures, bonus payments, number of aligned beneficiaries, and hospital affiliation. Data on MA enrollment were obtained from the 2013 to 2021 regional variation public use files,^3^ and information on the USPCC was obtained from the 2014 to 2021 MA rate calculation files.^4^ Because Medicare payment rates are updated annually, in part to reflect inflation, all costs and spending are reported in current year dollars. No discount rate was applied. The Brown University institutional review board deemed the project exempt because only publicly accessible, aggregate data were used. Informed consent was not possible for analysis. The study followed the Consolidated Health Economic Evaluation Reporting Standards (CHEERS) reporting guideline.

### Statistical Analysis

To estimate net savings of the MSSP in traditional Medicare, MSSP bonus payments overall and per aligned beneficiary were calculated for each performance year. Estimates of the impact of the MSSP were then extracted from 2 studies finding that the program was associated with reduced gross medical spending: a study by McWilliams et al^1^ published in 2018 (−$253.05 per beneficiary per year for physician-affiliated ACOs; –$49.48 for hospital-affiliated ACOs) and a study by the Medicare Payment Advisory Commission (MedPAC)^2^ published in 2019 (−$103.53 per beneficiary per year overall). These estimates were applied across the 2013 to 2021 period (eMethods in Supplement 1). Net savings to traditional Medicare were calculated by taking the difference between MSSP bonus payments with gross reductions in medical spending.

The association between the net impact of the MSSP and benchmark payments to MA plans was then assessed. The net impact of the MSSP per beneficiary in a given performance year was multiplied by the share of traditional Medicare beneficiaries in the MSSP. The product of these quantities was then multiplied by the number of MA beneficiaries whose payment was linked to traditional Medicare spending to estimate the effects on benchmarks in the following year (eTable 1 in Supplement 1).

## Results

The MSSP bonus payment per beneficiary increased gradually between 2013 ($85) and 2019 ($112) before increasing sharply after the COVID-19 pandemic in 2020 ($215) and 2021 ($194) (Table). The share of hospital-aligned beneficiaries increased from 61.2% in 2013 to 69.3% in 2021, and the share of traditional Medicare beneficiaries attributed to the MSSP increased from 10.9% in 2013 to 32.8% in 2021.

**Table.** Bonus Payments, Share of Beneficiaries Attributed to Hospitals, and Total Attributed Beneficiaries in the MSSP

MSSP performance year	Bonus payments per beneficiary, $	Share of beneficiaries in ACOs affiliated with a hospital vs physician-only ACOs, %	Total attributed MSSP beneficiaries	Share of TM beneficiaries attributed to MSSP, %
2013	85	61.2	3 675 263	10.9
2014	64	60.5	5 329 831	15.9
2015	89	65.8	7 270 233	21.7
2016	88	67.3	7 884 058	23.2
2017	87	67.1	8 992 886	26.6
2018	96	70.0	10 096 874	30.1
2019	112	70.7	9 997 705	30.2
2020	215	69.6	10 614 589	32.7
2021	194	69.3	10 124 325	32.8
Pre–COVID-19 average	91	67.7	7 606 693	22.6
Overall average	123	68.3	8 220 641	24.8

Abbreviations: ACO, accountable care organization; MSSP, Medicare Shared Savings Program; TM, traditional Medicare.

**Figure 1.**
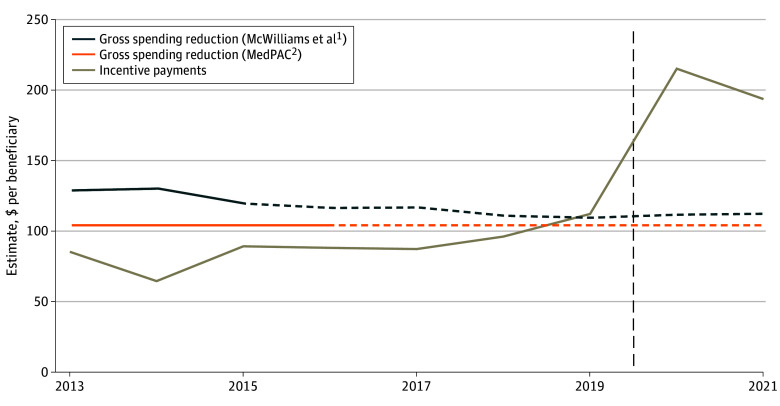
Estimates of the Reductions in Traditional Medical Spending and Incentive Payments per Beneficiary in the Medicare Shared Savings Program Dashed horizontal lines denote estimated effects occurring after the end of the study period; dashed vertical line denotes the onset of the COVID-19 pandemic. MedPAC indicates Medicare Payment Advisory Commission.

**Figure 2.**
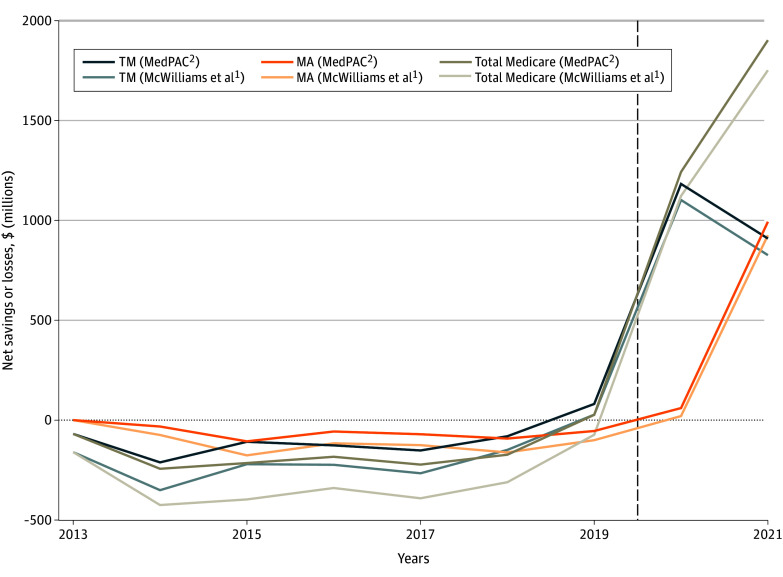
Estimates of Net Savings From the Medicare Shared Savings Program Values above 0 denote net losses to Centers for Medicare & Medicaid Services (CMS), while values below 0 denote net savings to CMS; dashed vertical line denotes the onset of the COVID-19 pandemic. MA indicates Medicare Advantage; MedPAC, Medicare Payment Advisory Commission; and TM, traditional Medicare.

Estimates derived from McWilliams et al^1^ indicate that spending reductions in traditional Medicare were larger than incentive payments between 2013 and 2018 before becoming smaller than incentive payments between 2019 and 2021 (Figure 1). Estimates derived from MedPAC^2^ followed a similar pattern. Together, this resulted in total net losses in traditional Medicare of $584 million based on estimates from McWilliams et al^1^ and $1.423 billion based on estimates from MedPAC^2^ (Figure 2).

Net savings from reductions to MA benchmarks accrued to CMS between 2014 and 2019 while net losses from increases to MA benchmarks occurred in 2020 and 2021 (Figure 2). Losses from MSSP-related increases to MA benchmarks totaled $191 million over the study period based on estimates from McWilliams et al^1^ and $640 million based on estimates from MedPAC^2^.

Summing net losses from traditional Medicare and MA, the MSSP was associated with total losses to CMS of $775 million based on estimates derived from McWilliams et al^1^ and $2.063 billion based on estimates derived from MedPAC.^2^ This represents approximately 0.028% of combined medical spending in traditional Medicare and MA over the study period.

## Discussion

Using frequently cited estimates of the impact of the MSSP, this study found that the MSSP was associated with net losses to CMS during performance years 2013 to 2021. To our knowledge, this study is the first assessment of the net savings of the MSSP in traditional Medicare after the COVID-19 pandemic and the first to assess the association of the MSSP with payment in MA. The finding that the MSSP was associated with net losses to traditional Medicare conflicts with other research that the MSSP was associated with net savings of approximately $250 million annually.^1^ While we found similar savings in the early years of MSSP implementation, our findings diverged in more recent years of MSSP implementation. The was the result of rising bonus payments to MSSP ACOs in the postpandemic period, the shift in MSSP beneficiaries toward hospital-aligned ACOs, and the incorporation of estimates of the impact of the MSSP on MA benchmarks.

### Limitations

The study was limited by the lack of evaluations of the consequences of the MSSP for gross spending in performance years 2017 to 2021 and inherent challenges projecting effect estimates of the MSSP in light of heterogeneous treatment effects. Effect estimates of the MSSP between 2017 and 2021 may have been higher than estimates from earlier periods as a result of a greater duration of exposure to the program (eTable 2 in Supplement 1) but may have been lower due to weaker performance among later entry cohorts.^1^ The net impact of these countervailing effects is ambiguous. In addition, 2 of the study years overlapped with the COVID-19 pandemic. This may have affected outcomes from the MSSP by diverting health systems toward pandemic-related issues and away from ACO priorities (eg, care management and reducing low-value care).^5^ In addition, CMS mitigated shared losses to MSSP ACOs during the pandemic and made some changes to benchmarks on the basis of spending related to COVID-19. However, higher bonus payments, rather than lower ACO losses, were primarily responsible for higher net bonus payments during the pandemic, as only a small share of ACOs were penalized over the duration of the MSSP in this study. These higher bonus payments resulted from lower spending during the pandemic and prospectively set benchmarks that were not adjusted to account for secular shocks, such as the COVID-19 pandemic.^5^ The study was also limited by missing information on hospital affiliation for 12.1% of beneficiaries, although our 2012 to 2015 estimate of hospital-aligned ACO beneficiaries (63.2%) is similar to that of McWilliams et al^1^ (59.7%). Total costs to CMS were underestimated as we could not account for administrative or opportunity costs of the MSSP.

Importantly, estimates of the effects of the MSSP used in this study likely represent an upper bound of savings in the program. Evaluation evidence shows that estimates of the MSSP are sensitive to the construction of treatment and comparison groups and sensitive to strategies used to address compositional changes in the clinicians and beneficiaries aligned with ACOs over time.^2,6,7^ McWilliams et al^1^ attempted to address bias from nonrandom alignment into the MSSP by disallowing assignment to ACOs on the basis of care provided in nursing facilities. However, this does not rule out other strategies used by ACOs to avoid attribution of high-spending physicians and patients. Research using lists of officially attributed beneficiaries and accounting for nonrandom attrition and compositional differences in patients and clinicians found smaller effects of MSSP than the McWilliams et al^1^ and MedPAC^2^ analyses described in this study.^6,7^

## Conclusions

This economic evaluation found that the MSSP was associated with net losses to CMS. Our results highlight important tensions in CMS’s voluntary ACO models. Generating savings in traditional Medicare requires finely tuned incentives that are sufficiently generous to encourage participation but sufficiently strict to generate savings. This is hard to accomplish over the long term and susceptible to unexpected events (like COVID-19) that undermine benchmark-setting assumptions. In addition, because of the link between traditional Medicare spending and MA benchmarks, losses from ACO models and other alternative payment models inflate payments in MA. Our findings highlight the budgetary importance of designing alternative payment models that generate net savings for CMS.

## Notes

Supplement 1.
eMethods.
**eTable 1.** Values for the Calculation of the Estimated Impact of the MSSP and on Benchmarks **eTable 2.** Average Duration of ACO Participation by Program Year

Supplement 2.
Data Sharing Statement

Supplement 3.
Retracted article with errors highlighted

Supplement 4.
Replacement article with corrections highlighted
