# Supplementary material for: Estimated Savings From the Medicare Shared Savings Program
Source: JAMA Health Forum. 2023 Dec 15;4(12):e234449. doi: 10.1001/jamahealthforum.2023.4449 (PMC10724775; doi:10.1001/jamahealthforum.2023.4449)
Supplement: Supplement 1. — eMethods. eTable 1. Values for the Calculation of the Estimated Impact of the MSSP and on Benchmarks eTable 2. Average Duration of ACO Participation by Program Year [file jamahealthforum-e234449-s001.pdf]

Supplementary Online Content

Ryan AM, Markovitz A. Estimated savings from the Medicare Shared Savings Program. *JAMA Health Forum*. 2023;4(12):e234449. doi:10.1001/jamahealthforum.2023.4449

eMethods.

eTable 1. Values for the Calculation of the Estimated Impact of the MSSP and on Benchmarks

eTable 2. Average Duration of ACO Participation by Program Year

This supplementary material has been provided by the authors to give readers additional information about their work.

© 2024 Ryan AM et al. *JAMA Health Forum.*

eMethods.

Calculating estimates of the effect of the MSSP on spending from McWilliams et al. and MedPAC

McWilliams et al. (2018) report separate estimates for physician-affiliated and hospital-affiliated ACOs separately for each entry cohort (2012, 2013, and 2014) across the duration of exposure to the program (3 separate years for the 2012 and 2013 cohorts; 2 years for the 2014 cohort). This results in a total of 8 separate effects for both hospitals and physicians (reported as per member per year and in percentage terms). Taking the unweighted average across these 8 estimates, we calculated the estimate to be -$252.35 per member per year for physician-only ACOs and -$49.15 for hospital-affiliated ACOs. We then calculated the weighted average of these two estimates in each performance year based on the share of patients in physician-only and hospital-affiliated ACOs in each of the MSSP.

MedPAC reported the impact of the MSSP as the difference in percentage point change in annual spending between 2012 and 2016 between: 1) beneficiaries aligned to an ACO in the 2013 cohort; 2) non-aligned beneficiaries in the same market area. While MedPAC reported several alternative estimates, we used the estimate that the MSSP was associated with a 1.7 percentage point reduction in this growth rate which was derived from a propensity weighted regression model. We used this estimate because it accounted for covariates in a regression framework. To convert this estimate into per member per year units, we first calculated the share of the 2013 entry cohort that lived in each US county. We then used these estimates to calculate the weighted market average of risk-adjusted and standardized Medicare spending in 2012 ($9822.42 per member per year) and 2016 ($10,226.64 per member per year). This amounted to a change in spending of 4.1 percentage point change in spending. If the change in spending was 1.7 pp less for ACOs, this would have amounted to a change of 2.4 percentage points between 2012 and 2016. This results in an average reduction in spending per member per year of $103.96.

Attributing hospital affiliation status to ACOs

The 2013-2017 public use ACO performance files did not contain information on whether a specific ACO had a hospital affiliation. Hospital affiliation began to be reported beginning in 2018. Nonetheless, many ACOs participated in the MSSP longitudinally. To address this issue, we first evaluated whether an ACO was affiliated with a hospital in any of the performance years in which it participated and then attributed this status across its duration of participation. For instance, if an ACO began in the 2017 performance year (and had missing information as to its hospital affiliation) but was indicated to have a hospital affiliation in the 2018 performance year, we would have attributed this affiliation to the 2017 performance year. After this procedure, hospital affiliation remained missing for 12.1% of beneficiaries.

Considering the impact of MSSP gross spending reductions on Medicare Advantage

There are two important elements that contribute to MA benchmark calculations: the projected national TM per capita spending for a given benchmark year (known as USPCC) and the ratio of lagged county level TM spending to national TM spending (known as the average geographic adjustment (AGA). County benchmarks are determined by multiplying the USPCC by the AGA,

resulting in different values for each county (which are subject to additional adjustment which we do not consider).

The MSSP will have little to no overall impact on the magnitude of MA benchmarks by virtue of changes to the AGA. Some context: according to the Congressional Research Service, since 2014, shared savings and penalties administered through CMMI have been incorporated into county-level spending estimates used in the AGA.[1] This will increase benchmarks in some counties and decrease benchmarks in others. Yet because this is a ratio, the net effect of these adjustments is approximately zero. The only way that payments related to shared savings and losses would affect aggregate benchmarks is if Medicare Advantage enrollment shifted between the period in which the AGA is calculated (e.g. 2010-2014 for benchmark year 2017) and the benchmark year. We have no evidence that this occurred and did not incorporate estimates of the effects of the MSSP on the AGA in our analysis.

Instead, the MSSP could affect the calculation of the USPCC. The USPCC is a prediction of national TM spending for a given benchmark year based on a time series of prior TM spending. To the extent that the MSSP changed net TM spending (inclusive of medical spending and incentive payments to ACOs), this will be reflected in the USPCC. This can be seen in how CMS calculates the USPCC. [2] In the 2024 rate announcement that was released in 2023, there is a line item labeled “Innovation model shared savings (Includes APM).” This would include incentive payments in the MSSP. This line item is positive for Part A and Part B in all years, demonstrating how alternative payment model incentive payments increase the USPCC.

As described in the Methods, we estimated the association between the net impact of the MSSP and benchmark payments to MA plans. The net impact of the MSSP per beneficiary in a given performance year was multiplied by the share of traditional Medicare beneficiaries in the MSSP. The product of these quantities was then multiplied by the number of MA beneficiaries whose payment was linked to traditional Medicare spending to estimate the effects on benchmarks in the following year.

eTable 1 shows several of the values used for this calculation. We will illustrate how we calculated the impact of the MSSP on benchmark changes in MA payment year 2021. In 2020, the McWilliams-derived estimate of the net impact of the MSSP on TM spending was the $104, and 32.7% of traditional Medicare beneficiaries were in the MSSP. Multiplying these together, the impact of the MSSP on the USPCC was $34 in 2020. Multiplying this by the number of MA beneficiaries whose payment is indexed to the USPCC in 2021 (27,219,996) results in an increase in MA payments of $926 million in 2021.

References

1. Morgan PC. “Medicare Advantage (MA)–Proposed Benchmark Update and Other Adjustments for CY2020: In Brief.” Congressional Research Service; 2019. https://crsreports.congress.gov/product/pdf/R/R45494/3.

2. The Centers for Medicare and Medicaid Services. Reconciliation of Non-ESRD FFS USPCC Claims with Source Data, 2019-2021. Last updated September 6, 2023. Accessed January 4, 2024. https://www.cms.gov/files/document/non-esrd-uspcc-reconciliation-2024-rate-announcement.pdf

© 2024 Ryan AM et al. *JAMA Health Forum.*

eTable 1. Values for the calculation of the estimated MSSP MA benchmarks

Year	USPCC^	TM benes in MSSP, %	Number of MA benes*	Estimates derived from McWiliams et al.			Estimates derived from MedPAC		
				MSSP impact on net TM spend^	MSSP impact on USPCC^	Impact on MA paymentsǂ	MSSP impact on net TM spend^	MSSP impact on USPCC^	Impact on MA paymentsǂ
2013	9,471	10.9	-	-44	-5	0	-19	-2	0
2014	9,751	15.9	15,634,897	-66	-10	-75	-40	-6	-32
2015	9,226	21.7	16,837,948	-30	-7	-177	-15	-3	-106
2016	9,603	23.2	17,661,028	-28	-7	-116	-16	-4	-57
2017	9,902	26.6	19,037,548	-30	-8	-125	-17	-4	-71
2018	10,173	30.1	20,565,664	-15	-4	-162	-8	-2	-92
2019	10,693	30.2	22,577,684	3	1	-101	8	2	-55
2020	11,290	32.7	24,705,856	104	34	20	111	36	60
2021	11,701	32.8	27,219,996	82	27	926	90	29	993

TM: traditional Medicare; MSSP: Medicare Shared Savings Program
* In non-cost MA plans whose payment is subject to USPCC
^$ per beneficiary
ǂ $ in millions

© 2024 Ryan AM et al. *JAMA Health Forum.*

eTable 2. Average duration of ACO participation by program year

	Duration		
Year	Mean	95% CI, lower limit	95% CI, upper limit
2013	1.0	1.0	1.0
2014	1.7	1.7	1.8
2015	2.3	2.2	2.4
2016	2.9	2.7	3.0
2017	3.3	3.1	3.4
2018	3.6	3.3	3.8
2019	4.4	4.2	4.7
2020	4.5	4.2	4.8
2021	5.4	5.1	5.7

© 2024 Ryan AM et al. *JAMA Health Forum.*
