# Supplementary material for: Estimated Savings From the Medicare Shared Savings Program
Source: JAMA Health Forum. 2023 Dec 15;4(12):e234449. doi: 10.1001/jamahealthforum.2023.4449 (PMC10724775; doi:10.1001/jamahealthforum.2023.4449)
Supplement: Supplement 2. — Data Sharing Statement [file jamahealthforum-e234449-s002.pdf]

Data Sharing Statement

Ryan. Estimated Savings From the Medicare Shared Savings Program. *JAMA Health Forum*. Published December 15, 2023. doi:10.1001/jamahealthforum.2023.4449

Data
Data available: Yes
Data types: Data (not involving human participants), Data dictionary
How to access data: Github
When available: With publication

Supporting Documents
Document types: Statistical/analytic code
How to access documents: Github
When available: With publication

Additional Information
Who can access the data: anyone
Types of analyses: anything
Mechanisms of data availability: no permissions required
